# Effects of music on the spatial cognitive performance, growth performance and stress response of sheep

**DOI:** 10.5713/ab.24.0416

**Published:** 2025-03-31

**Authors:** Jingyi Tu, Changqing Shen, Ruiling Lei, Lang Li, Shicheng Wang, Siqi Peng, Xiong Xiao, Yongju Zhao, Xiaoyan Qiu

**Affiliations:** 1 Chongqing Key Laboratory of Herbivore Science, College of Animal Science and Technology, Southwest University, Chongqing, China; 2 College of Veterinary Medicine, Southwest University, Chongqing, China

**Keywords:** Cognitive Performance, Growth Performance, Music, Sheep, Stress Response

## Abstract

**Objective:**

This study aimed to investigate the effect of two music types on spatial cognitive ability, growth performance, and psychological cortisol response of sheep.

**Methods:**

The “Y-spatial and Reversal Test”, as the internationally recognized behavioral test for spatial cognitive function of large animals, was applied in this study to evaluate the effects of music on spatial cognitive performance of sheep. The average daily gain (ADG), average daily feed intake, ratio of feed to gain (F/G), and the cortisol release in saliva and plasma were analyzed to evaluate the effect of music on growth performance and stress response of sheep.

**Results:**

The music of “Annie’s Wonderland” (55 dB) could make sheep establish the correct spatial reversal recognition more quickly, while the music of “Days of Youth Waltz” (55 dB) made sheep more restless and affected their performance in left-right spatial reversal recognition. The ADG of sheep in the “Annie’s Wonderland” (55 dB) group was significantly higher than that of the control group, and the F/G of the “Annie’s Wonderland” group was significantly lower than that of the control group (p<0.05). While the ADG in the “Days of Youth Waltz” (55 dB) group were significantly lower and the F/G was significantly higher than that of the control group (p<0.05). The salivary cortisol secretions of sheep in the “Annie’s Wonderland” (55 dB) group were significantly lower on Day 7, Day 14 and Day 21 than that of the control group (p<0.05) and the plasma cortisol concentration at the peak (20 min after adrenocorticotropic hormone injection) was also significantly lower than the control group.

**Conclusion:**

The music of “Annie’s Wonderland” (55 dB) could improve the spatial cognitive fuction, increase the ADG and lower the cortisol secretion in sheep, while the music of “Youth Waltz” (55 dB) is not recommended since it may negatively impact animal welfare.

## INTRODUCTION

Music is a kind of art which is used to express mood and emotions. Studies have shown the effects of music therapy on the physiology and psychology of humans [1]. The effects of music on behaviours and growth performance have been studied in different animal species including birds [2,3], chimpanzees [4], elephants [5], rats [6], dogs [7], cats [8,9], pigs [10], equine [11] and cattle [12]. Most of these studies suggested that the music exposure may benefit animals by decreasing the levels of stress and increasing their growth performance while other studies showed that the music has no effect [7]. Nevertheless, there has been no any reported study carried out to determine the effects of music on behaviours, growth performance and stress response in sheep.

Recent behavioural studies on sheep were much focused on their cognitive behaviour which refers to the ability to learn and establish the association between stimuli, actions and outcomes followed by an appropriate decision when they are faced with a challenge or changes in the environment [13]. Studies suggested that sheep have cognitive discrimination and learning ability [13–15]. Researchers from the University of Cambridge had demonstrated that sheep had the ability to recognize and memorize not only for left-right direction through spatial discrimination and reversal learning but also for colours and shape through visual discrimination and reversal learning [13]. The left-right spatial cognitive performance of white cashmere goats and Chinese merino sheep using these two cognitive behavioural tests has been recently explored and revealed that both sheep and goats have spatial cognition [16]. Cognition of sheep has been reported to be associated with the temperament [15], which itself greatly influences the reproduction and production of sheep [16–19]. Therefore, the cognitive performance of sheep can reflect the production and welfare of sheep, however, there is limited studies about the effect of music on sheep cognitive performance.

Therefore, this study, for the first time, investigated the effect of the two music on the spatial cognitive performance, growth performance and stress response of sheep, which provided a theoretical basis for the application of music in improving the production and welfare of sheep.

## MATERIALS AND METHODS

### Animals

This experiment was approved by Institutional Animal Care and Use Committee of Southwest University (SWU). The code for laboratory animal operation is SWU_LAC-2022100099. In this experiment, total of 80 Corriedale ewes aged 3 months old with body weight of 22.5±2.0 kg were selected. They were naive to cognitive testing, and had no pretraining before this study. The ewes were housed at the farm in a paddock-feedlot system where they had free access to feed and water for the experimental period before and during cognitive testing. The ewes were reared in the stall-feeding condition during the tests about the growth performance and cortisol secretion. The three groups of ewes are reared in three seperate walled stalls to avoid the music interference with each other. Each stall is 7 meters long and 6 meters wide. The daily feeding includes fresh forage and mixed concentrate. The amount, proportion and composition of the feed given to each group were the same. The composition of fresh forage consists of 10% alfalfa, 20% clover and 70% ryegrass; the concentrate is purchased commercially prepared, the ingredients include corn, bran and soybean meal.

### Effects of music on the cognitive performance of ewes

#### Habituation

Two weeks before testing, ewes were habituated to the test apparatus. During habituation, ewes were allowed to explore the testing area and eat feeds they found placed in the buckets. This aimed at making the tested ewes to be familiar with the apparatus and testing situation, so that the subsequent tests could accurately reflect the actual cognitive levels (to prevent the tested ewes from being stressed by the novelty of the device, which may interfere and affect the establishment of correct judgment and cognition). Ewes were naive to cognitive testing, and had no any training before they were used in this experiment.

#### Experimental design

The “Y-spatial discrimination and reversal test” was used to screen the individuals with basically the same level of cognition according to the behavioural test scores (the total time taken and the number of choice errors). 15 tests were carried out in the Y-spatial test, while 20 tests in the reversal test. We conducted only 3 trials for each ewe on the first day, given the evidence that isolation in ewes is resulted into stress. Five trials were conducted on the 2nd day while 7 trials were done smoothly on the 3rd day of experiment. In each day of the experiment, the same number of trials was performed in all ewes. Ewes were considered to have met the criterion for learning the discrimination if they made 4 consecutive correct choices (i.e. the number of error choice is 0).

Thirty ewes with similar spatial cognition test scores (eliminated the factor of individual cognitive difference) were selected for the music stimulation test that follows thereafter. Then, they were divided into three groups of 10 ewes each: the control group; the group listening “Annie’s Wonderland” (55 dB; Bandari [Annie’s Wonderland] https://c6.y.qq.com/base/fcgi-bin/u?__=xWw2Q9bGhOtL); and the group listening “Days of Youth Waltz” (55dB; Dick Rodgers [Days of Youth Waltz] https://c6.y.qq.com/base/fcgi-bin/u?__=DL1HcDCGhdGG). Each group was subjected to the same “spatial discrimination and reversal test” to assess the effect of the music on the spatial cognitive performance of ewes.

#### Testing device and method

**Figure 1:**
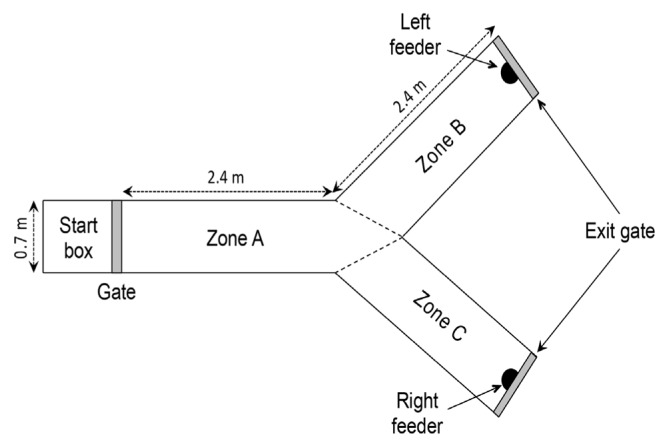
Scheme of the Y-maze apparatus (based on Qiu et al [15]). The maze is divided in 3 zones (A, B and C). Feeders, either empty or containing the food reward, were placed at the end of the two arms (zones ‘B’ and ‘C’). Every side of the Y-maze was covered with hessian to isolate the animal from external visual stimuli. The food reward was always placed in a feed bucket on one arm of the maze, with an empty feed bucket placed on the other arm. The experiment started by allowing the ewes to pass through the starting gate into the run (Zone A), from which it had to make a choice within 2 minutes toward the left (Zone B) or right (Zone C) arm of the maze. The time taken by the ewes to find and eat the food reward as well as the number of choice errors were recorded.

The “Y-spatial discrimination and reversal test” is an internationally recognized and widely used behavioural test for spatial cognitive function of large animals including sheep and monkeys [13–15]. If the animal can perform the task well in the test, it means its spatial cognitive function is normal, otherwise it is impaired. The Y test device used for spatial cognition test is built as shown in Figure 1 below, and the test method is based on our previous study [15].

The experiment started by allowing the ewes to pass through the starting gate into the run (A), from which it had to make a choice within 2 minutes toward the left (B) or right (C) arms of the maze. The animals were free to move around the maze accessing different zones. We recorded the time taken by the ewes to find and eat the food reward as well as the number of times the ewes crossed the boundaries into different zones until it found and started eating the reward. Half of each treatment group was trained to find the reward on one side of maze and the other half trained to find it on the other side. A ewe was considered to have achieved spatial discrimination when it had made 4 consecutive correct choices (running straight toward the correct side; error choice = 0). After that, the animals were tested for reverse spatial discrimination by swapping the location of the reward. The same criterion was used to assess the success in the reversal discrimination. The differences of the number of errors and the time to eat the food among groups were analyzed.

### Effects of music on the growth performance of ewes

This experiment was carried out over 21 days where music was played from 8:00 to 11:00 am and 2:00 to 5:00 pm every day for each group with a single song cycle. The music of “Annie’s Wonderland” and the music of “Days of Youth Waltz” were played in the two music groups. The average daily gain (ADG), average daily feed intake (ADFI) and feed/gain (F/G) were calculated. Ewes of each group were weighed at the beginning and the end of the experiment for the purpose of calculating the ADG. To calculate the ADFI, nine ewes were used for each group, with three replicates per group (three ewes per replicate). Each group of ewes were reared in the seperate walled stall to avoid the music interference with each other. Within each stall, half walls were built to divide the stall into three small stalls, with three ewes in each small stall, and there is a trough in each small stall, so there are three replicates for each group. The daily feed intake was calculated from the amount fed given minus the amount left over. The amount of the feed given to each group per day was the same. The feed were given into the trough of each group at 7 am, and the left over in the trough of each group were weighed at 9 pm.

### Effects of music on the salivary cortisol secretion of ewes

This experiment was also carried over 21 days, with music played from 8:00 to 11:00 am and 2:00 to 5:00 pm per day. The gauze strips were hunged above the trough for the sheep to chew freely. The gauze strips were cut off and placed in a syringe to squeeze out the saliva at 6 pm on the collecting days. In order to minimize the disturbance to the animals, saliva was collected on Day 1, 7, 14, and Day 21. The cortisol concentration was then measured by the use of saliva cortisol detection kit (MP Biomedicals, Shanghai, China).

### Effects of music on the plasma cortisol secretion of ewes

This experiment was carried in the end. The cortisol, as the final effector of hypothalamic-pituitary-adrenal (HPA) axis, is known to play an important role in stress responses, hence it is frequently used as an indicator of the physiological response to stressors [20]. The physiological adrenal responsiveness was measured with an adrenocorticotropic hormone (ACTH) challenge. The endogenously driven secretion of cortisol was blocked by using dexamethasone, a synthetic glucocorticoid that acts at hypothalamic level to inhibit the secretion of ACTH [21,22]. The release of endogenous ACTH was prevented with a purpose of getting a low background concentration of cortisol. With a low background concentration of cortisol, tests using exogenous ACTH to elicit a cortisol response from the adrenal glands become far more predictable, allowing robust comparison of a cortisol response among groups of ewes.

Every ewe was injected with dexamethasone (0.125 mg/kg i.v.) to suppress the endogenous release of ACTH, and then injected with the saline or ACTH 90 min later (time = 0). Half of the sheep from each group received a 3 mL intravenous injection of either saline (vehicle control) or saline containing 0.2 IU ACTH. Blood (3 mL) was sampled at 90, 60, 30 and 0 min before injection of saline or ACTH and then every 20 min for 3 h after injection.

A day before the experiment, cannulation of the jugular vein for serial blood collection was done as follows:

*Animal preparation*: a. Wool was clipped to expose the skin over the jugular vein, providing an area big enough to allow easy visualization and access to the jugular vein;

b. Local anaesthetic cream was applied (lignocaine 25 mg/g, prilocaine 25 mg/kg) to the area and the time of application was recorded;

c. The animal was then sedated with ilium xylazil (0.1 mg/kg i.m.);

d. Approximately 30 min after application of the local anaesthetic cream, the animal was properly restrained in a standing position.

*Site Preparation*: a. On cannulation trolley, all the needles, tubing and equipment were placed in 70% ethanol for disinfection;

b. The shaved area was scrubbed with betadine surgical scrub;

c. The vein was clearly located by occlusion plus visualization of rise and fall with presure and release, before moving on to the next step;

d. A small incision was made on the skin above the vein using a scalpel blade;

e. A 13G luer lock needle was inserted into the jugular vein and the cannula tubing was quickly connected.

f. A 3-way tap was attached to the end of the cannula.

g. Blood flow was verified using a syringe filled with sterile, heparinised (40 IU/mL) 0.9% saline; the lumen of the cannula was rinsed by injection of 2 to 3 mL heparinised saline.

h. A piece of fabric tape was wrapped around the tubing at the point where it enters into the skin, leaving two ‘wings’ on either side that could be sutured to the skin to secure the cannula in place.

i. Another piece of fabric tape was wrapped around the junction between the tap and tubing to reduce the risk of twisting and perforation of the tubing.

j. The cannula and tap were laid on the neck of the sheep and 2 to 3 layers of packing tape were wrapped around the neck to ensure that the cannula and tap were covered while ensuring that breathing is not restricted.

k. At the end of the experiment, the cannula was removed and the cannulation site checked for bleeding.

Samples were immediately transferred into plastic tubes containing 50 IU heparin and polystyrene granules, and then centrifuged at 2,000 g for 10 min at room temperature. The plasma was separated and stored at −20°C for futher assay. Plasma cortisol concentrations were analysed using the Immuchem Coated Tube Cortisol^125^I RIA kit (MP Biomedicals). The limit of detection was 1.7 ng/mL. Quality control samples (30.5 ng/mL and 13.5 ng/mL) were used to estimate inter-assay and intra-assay coefficients of variation.

### Statistical analysis

Data were analyzed using SPSS (16.0 V). A two-factor analysis of variance (ANOVA) for repeated measurements in the General Linear Model was used to analyze the data with trials as the repeated measure, music as a factor and order as blocking factors. Prior to analysis, all data were assessed for normality using the Shapiro-Wilk test and for homogeneity of variance using Bartlett’s test. Where data were normally distributed and where variance was homogenous, the data were compared using Tukey’s post-hoc tests. The data for total time (s) in the spatial discrimination test was transformed using a cosine function and the other sets of data (total time in the spatial reversal test, number of errors in the spatial and reversal tests) were transformed using Blom’s formula in the transformation program in the software. Given the observed differences in standard deviations across the conditions, Maulchy’s test of sphericity was used to test whether variances and covariances of the within-subject variables were equal. If the test was significant (p<0.05) for each factor and for the interactions, it indicated a violation of the ANOVA assumption for compound symmetry, and a more conservative Greenhouse-Geisser F-test was used. For the cortisol response, data were analyzed using ANOVA for repeated measurements of the General Linear Model in SPSS, with time as the repeated measure and music as the factor. Differences were considered significant at p<0.05 and highly significant at p<0.01.

## RESULTS

### Effects of music on the spatial cognitive performance of ewes

**Figure 2:**
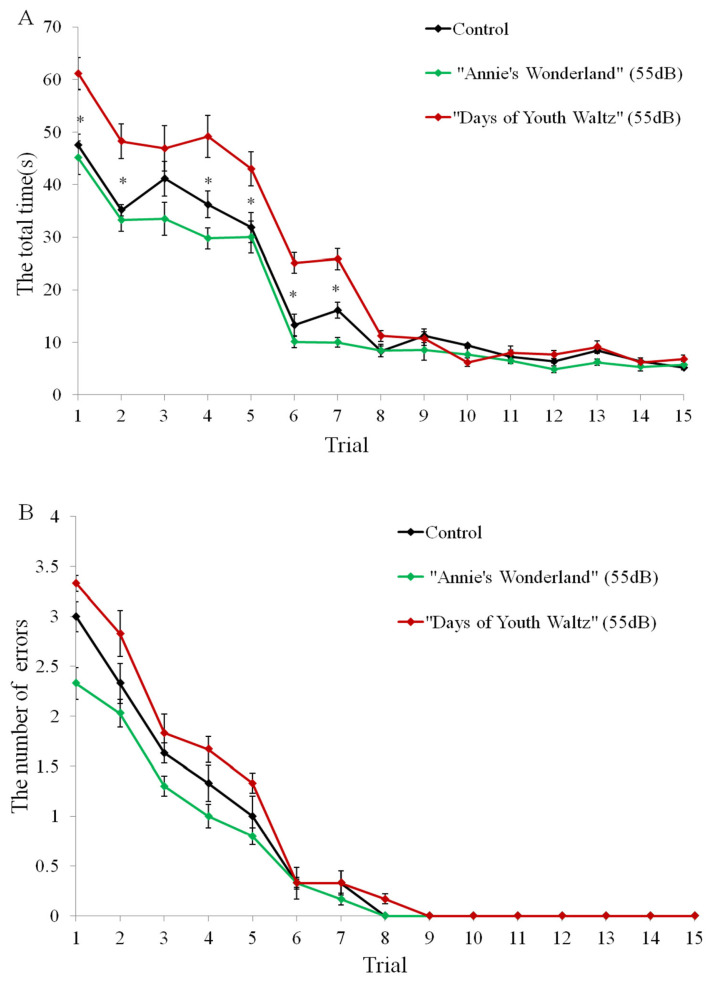
The behavioural results of the three groups in the Y-spatial test. A. The total time (seconds) taken to find and eat the food reward. B. The number of choice errors made before they found and eat the food reward. The animal was considered to have achieved spatial discrimination when it had made 4 consecutive correct choices (running straight toward the correct side; the number of errors = 0). After that, the animals were tested for spatial reversal discrimination by swapping the location of the reward (Figure 3). Mean±standard error; N = 10. * p<0.05.

There was a music effect (F_2, 447_ (sphericity assumed) = 3.517; p = 0.035) for the time (s) taken to complete the Y-spatial task. Ewes in the music of “Days of Youth Waltz” took more time in trials 1, 2, 4, 5, 6, 7 to complete the task than the control group (p<0.05; Figure 2A; Supplement 1). And there was an effect of trial within music (“Annie’s Wonderland”: F_14,135_ = 5.208, p = 0.033; “Days of Youth Waltz”: F_14,135_ = 6.081, p = 0.025) in both groups, with less time taken to complete the task over the trials. There was no music effect (F_2, 447_ [Greenhouse-Geisser] = 1.376; p = 0.205) and no interaction between time (trial) and music (F_14, 435_ [Greenhouse-Geisser] = 2.361; p = 0.071) for the number of choice errors in the test. There were no differences between groups in the number of error choices (p>0.05; Figure 2B). In Trial 8, ewes in the music of “Annie’s Wonderland” reached the learning criterion (error choice = 0) because they ran straight toward the correct side (Figure 2B), whereas ewes in the music of “Days of Youth Waltz” reached the learning criterion by Trial 9 (Figure 2B).

**Figure 3:**
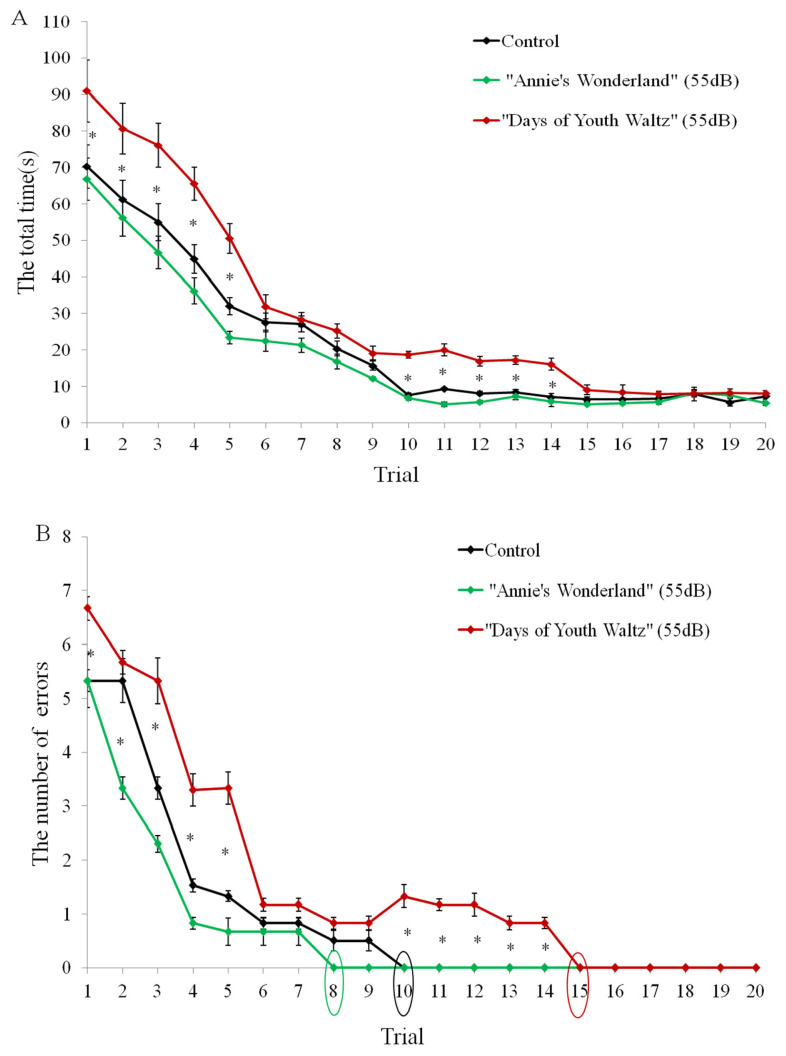
The behavioural results of the three groups in the Y-spatial reversal test. A. The total time (seconds) taken to find and eat the food reward. B. The number of choice errors made before they found and eat the food reward. The animal was considered to have achieved spatial reversal discrimination when it had made 4 consecutive correct choices (running straight toward the correct side; the number of errors = 0). Mean±standard error; N = 8. * p<0.05.

Two ewes from each group failed to reach the criterion for the reverse spatial discrimination test, so we analyzed the data for 8 ewes per group. There was an effect of music (F_2, 477_ [Greenhouse-Geisser] = 6.423; p = 0.026) for the time (s) taken to complete the reverse task. Sheep in the music of “Days of Youth Waltz” took more time in the first 5 trials and also during the middle trials 10–14 to complete the reverse task than the control group (p<0.05; Figure 3B; Supplement 2). And there was an effect of music (F_2, 477_ [Greenhouse-Geisser] = 7.569; p = 0.023) and interaction between time (trial) and music (F_19, 460_ [Greenhouse-Geisser] = 5.646; p = 0.031) for the number of error choices in the reverse test. There were differences in the number of errors in Trials 1, 3, 4, 5 and 10 to 14 (p<0.05; Figure 3B). The ewes in the music of “Days of Youth Waltz” made more error choices than the control group (p<0.05; Figure 3B). Ewes in the music of “Annie’s Wonderland” learned running straight to the correct side with no error by Trial 8, two trials earlier than the control group (Figure 3B). Ewes in the music of “Days of Youth Waltz” reached the learning criterion by Trial 15, five trials later than the control group (Figure 3B). Therefore, the music of “Annie’s Wonderland” tends to make ewes learned more quickly, while the music of “Days of Youth Waltz” made ewes learned more slowly.

**Figure 4:**
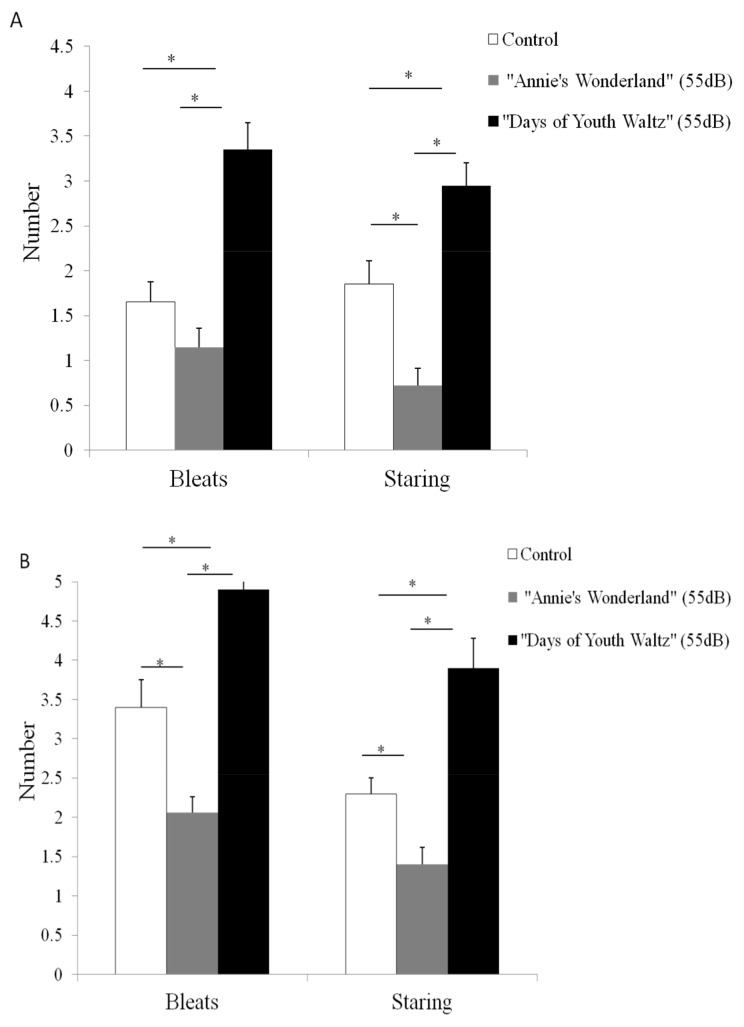
The behaviors of the three groups in the tests. A. The number of bleats and starings during the Y-spatial test. B. The number of bleats and starings during the Y-spatial reversal test. Mean±standard error. * p<0.05.

For all the three groups, the number of bleats and starings during the reversal spatial discrimination test (Figure 4B) were more than the spatial discrimination test (Figure 4A), indicating that the reversal discrimination test made ewes exposed to a more challenging environment (sheep had to go through a process of identifying mistakes in the initial test and correcting, and then developing reversal cognition in the opposite direction during the reversal spatial discrimination test). In the “Y-spatial test”, the number of starings in the “Annie’s Wonderland” group were significantly lower than that in the control group, while in the “Y-spatial reversal test”, both the number of bleats and starings in the “Annie’s Wonderland” group were significantly lower than that in the control group. In both tests, the number of bleats and starings in the “Days of Youth Waltz” group were significantly higher than that in the control group, suggesting that the music of the “Days of Youth Waltz” affected the behaviour of ewes and made them to be more restless.

### Effects of music on growth performance of ewes

**Figure 5:**
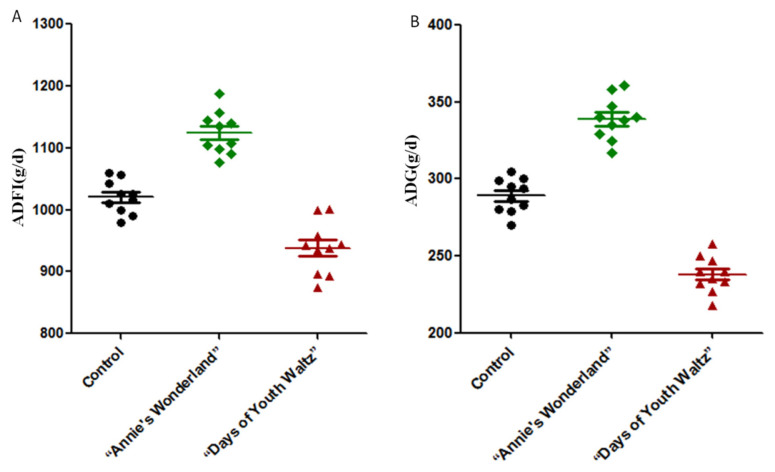
The growth performance with all individual data of the three groups. A. The individual data of ADFI (average daily feed intake) of the three groups. B. The individual data of ADG (average daily gain) of the three groups.

The ADG of sheep in the “Annie’s Wonderland” (55 dB) group was significantly higher than that in the control group (p<0.05) (Table 1; Figure 5), and the F/G of the “Annie’s Wonderland” group was significantly lower than that of the control group (p<0.05). While the ADG in the “Days of Youth Waltz” (55 dB) group were significantly lower and the F/G was significantly higher than that in the control group (p<0.05). As a result, the music of “Annie’s Wonderland” (55 dB) could increase the ADG of ewes. The “Days of Youth Waltz” is not recommended because ewes in this group had lower feed intake as well as slower growth rate.

### Effect of music on salivary cortisol and plasma cortisol concentration of ewes

**Figure 6:**
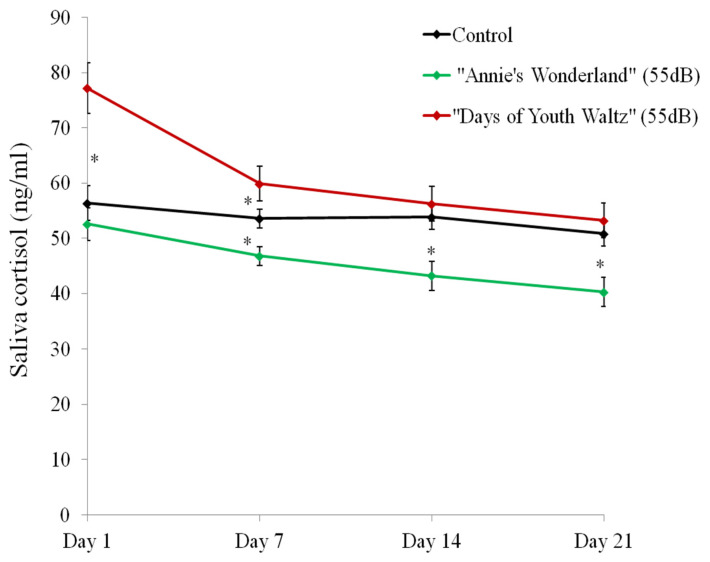
The salivary cortisol secretions of the three groups on Day 1, 7, 14, and 21 (mean±standard error; N = 10). * p<0.05.

**Figure 7:**
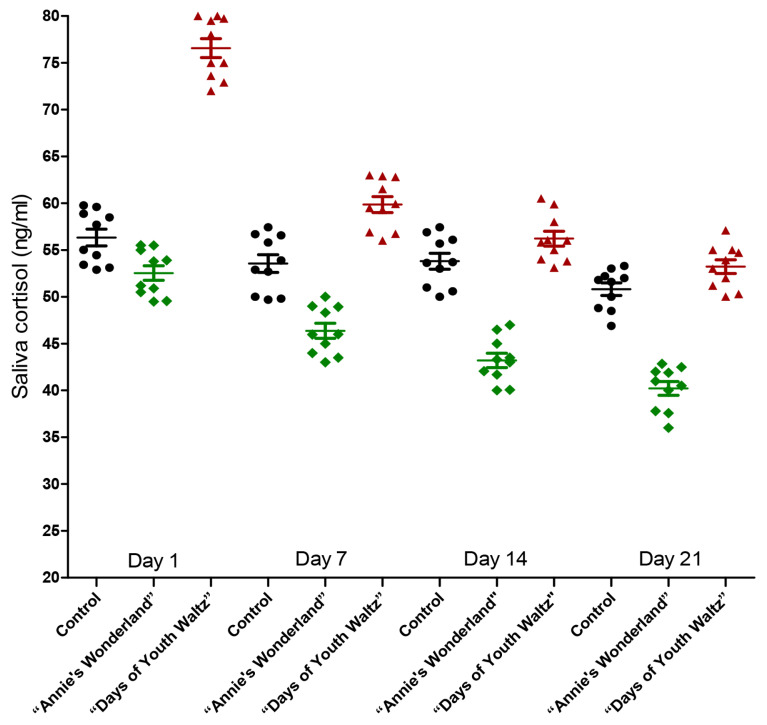
The individual data of salivary cortisol secretions of the three groups on Day 1, 7, 14, and 21.

The salivary cortisol secretion of the “Days of Youth Waltz” group on Day 1, 7, 14 and 21 was significantly higher than that of the “Annie’s Wonderland” group (p<0.05) (Figures 6, 7).

The cortisol levels lowered over time in both music groups. With exception to Day 1, the salivary cortisol secretion for the “Annie’s Wonderland” group was significantly lower than that of the control group (p<0.05) on Day 7, Day 14 and Day 21.

**Figure 8:**
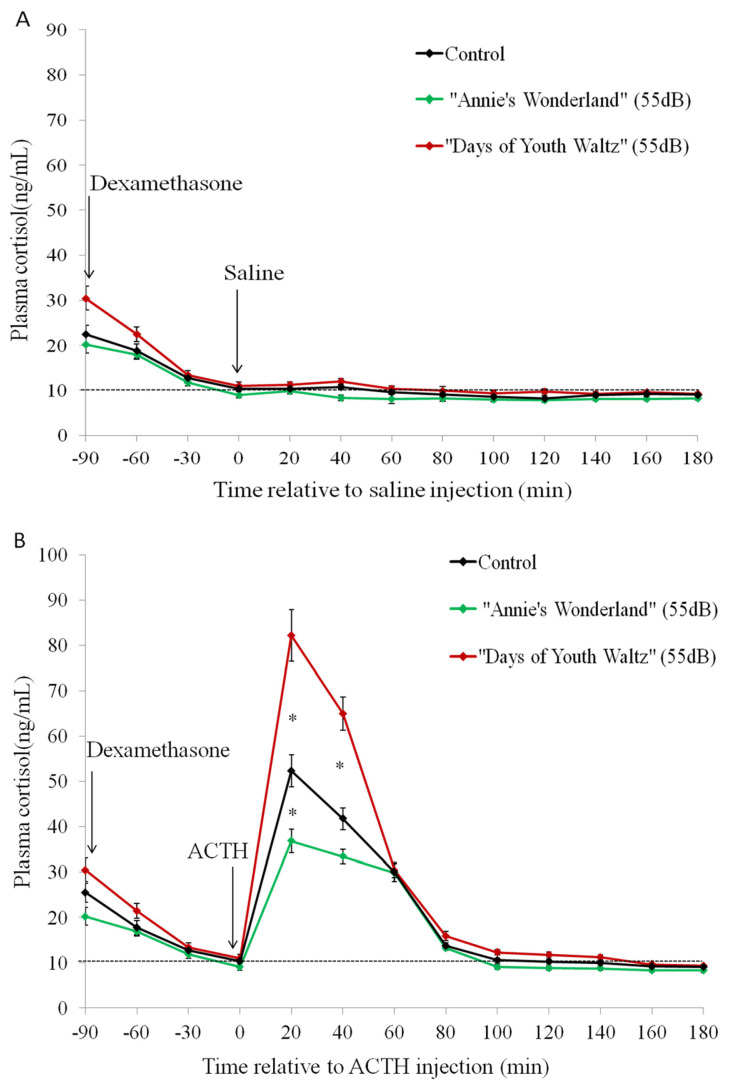
The plasma cortisol concentrations before and after intravenous injection of saline (A) and ACTH (B) in three groups of sheep. The dashed lines represent baseline of cortisol concentrations. * p<0.05. ACTH, adrenocorticotropic hormone.

The plasma cortisol test (Figure 8) showed that in the first hour after ACTH injection, sheep in the “Days of Youth Waltz” group had significantly higher plasma cortisol secretion while the concentrations at 20 min and 40 min after ACTH injection were significantly higher than those in the control group and the “Annie’s Wonderland” group. The plasma cortisol concentration at the peak (20 min) of the “Annie’s Wonderland” group was significantly lower than that of the control group, this indicate that the music of “Annie’s Wonderland” could lower the psychological cortisol response under a certain stimulation or pressure.

## DISCUSSION

### Effects of music on cognitive performance

There are currently very few studies on cognitive performance in large animals, particularly in sheep, and the majority of studies on cognitive behaviours in animals are focused on rats and mice. And the present studies about the effects of music on livestock are focused on their welfare and productive performance, while there are few studies on the influence of music on the cognitive behaviours of large animals, and the study about the effect of music on the cognitive performance and welfare of sheep is deficient. Therefore, this study is for the first time to explore the effect of two music on the spatial cognitive performance, the productive performance and the stress response in sheep.

Compared to the control group, the music of “Annie’s Wonderland” could make the ewes establish the correct spatial reversal recognition faster, while the music of “Days of Youth Waltz” made the ewes establish the correct spatial reversal recognition more slowly. These results are similar to what was reported in a study of mice by Hu et al where the music could influence the behaviours in the spatial discrimination test [23]. The effect of music on cognitive performance is probably mediated by reduced stress or general calmness [24]. This effect can be reflected through the behaviors (bleating and staring) (Figure 4). Ewes in the “Days of Youth Waltz” group showed more restless with higher salivary cortisol secretion at Day 1 and Day 7, and higher cortisol response at the peak in ACTH stimulation test.

### Effects of music on growth performance

There are many studies about the effects of music on the productive performance in birds [2,3] chimpanzees [4], elephants [5], rats [6], dogs [7], cats [8,9], pigs [10,25,26], equine [11], cattle [12], mice [27] and chicken [28], but few has been done in sheep. This study showed that the music of “Annie’s Wonderland” could increase the ADG, while the music of “Days of Youth Waltz” made ewes more restless and affected their ADFI, ADG and F/G. This finding is similar to what was reported in other studies in various species [12,25,27,28]. The study of long white piglets showed a 7.74% increase in the average resting time of the piglets in the group playing “Serenade” for 3 hours a day, 5.26% decrease in the active time and significantly less weaning stressful behaviour as compared to the control group [25]. Additionally, the ADG increased by 10.07%, and the feed utilization rate increased by 5.76% in the similar study [25]. Another study also showed that playing music in the environment significantly increased the weight gain of female rats [27]. In chickens, Huang et al found a significant increase in ADG of broilers after the music was played [28]. In cattle, the study showed that playing the classical music produced an increase in the milk production [12].

### Effects of music on stress response in animals

The cortisol, as the final effector of HPA axis, is known to be used as an indicator of the physiological response to stressors [20,29,30]. The physiological adrenal responsiveness was measured with an ACTH challenge test [31]. At present, there are studies about the relationship between music and stress on mice or rats [23,27], pigs [10], dogs [7] and cats [9], but no such studies have been done in sheep.

In this study, the plasma cortisol concentrations in all 3 groups of ewes reached the peak very quickly (20 min after injection of a low dose of ACTH (3 μg/ewe)), then decreased rapidly after 20 min, and returned to baseline values 2 h after ACTH injection, which is similar to what was reported in previous studies in sheep [18,22]. Conversely, other studies with sheep reported a longer duration in the cortisol response to ACTH that reached the peak value 60 min after ACTH and decrease thereafter [32,33]. This difference between studies can be explained by the dose-dependency of the cortisol response to ACTH stimulation [34,35]. In the other studies, the dose was 10 μg/kg or 500 μg per sheep [32,33], both of these doses being higher than 3 μg/sheep that was used in this study even if all of these doses are within the physiological range. The low dose of ACTH (0.1 to 5 μg) have been compared and it was found to be potent with ability to rapidly stimulate cortisol secretion to peak levels 20 min after ACTH administration, consistent with our observations. Similar results have been reported in studies on calves [36], pigs [37] and dogs [38]. In general, the cortisol peak can be reached earlier with the use of lower doses of ACTH than with higher doses [39]. Generally, the dose of ACTH that was used in this study seems to be the most appropriate for testing adrenal responsiveness.

The findings of this study suggested that playing the music of “Youth Waltz” could increase the salivary and plasma cortisol secretion, an indication that this music tends to make ewes to have a higher psychological response when exposed to a stressor, therefore this music is not recommended since it may negatively affect animal welfare. On the other hand, ewes in the “Annie’s Wonderland” group had lower cortisol response at the peak. These results are similar to those reported in other studies on various species. Hampton et al concluded that the music may benefit cats by decreasing the stress levels and increasing the quality of care in veterinary clinical settings [9], while Bowman et al suggested that classical music could reduce stress in kennelled dogs [7]. Wang et al showed that “Serenade” music can reduce the cortisol level of mice and effectively relieve their stress [27]. In this study, the effect of music on the psychological cortisol response can also partially support the theory behind the influence of the music on cognitive performance and productive performance of ewes.

### Effects of different music types

Studies have shown that the types of music could influence the effect of music on animals. Compared to the lively-type music like heavy, upbeat or rock music with fast-rhythm, the soothing-type music like the light, soft or classical music with slow-rhythm had a more positive effect on animal’s behaviours and productive performances. The study in mice by Hu et al [40] found that the soft music (Butterfly Lovers Violin concerto) could significantly improve the spatial memory ability of Kunming mice. Wells et al [41] showed that compared with modern upbeat music, the classical music can reduce barking behavior and increase resting behavior of dogs. Yu [42] found that in the environment of soothing music -”Annie’s Wonderland”, the weaned piglets had a significant increase of feeding behaviour, a reduction of the fighting and aggressive behavior and an improvement of the welfare level. Newberry [43] pointed out that the fast music (rock music) significantly reduced the daily gain of pigs and increased the ratio of feed to gain. Bowman et al suggested that classical music could reduce stress in kennelled dogs [7]. Wang et al. showed that playing “Serenade” (a soothing music) can reduce the cortisol level in mice [27].

Therefore, the effects of music on animal behavior, productive performance and welfare are related to the type of music played. However, there is very limited study about the effect of music types in sheep, so this study, for the first time, investigated the effect of two music types-the music of “Annie’s Wonderland” (a kind of soothing music) and the music of “Days of Youth Waltz” (a kind of lively music) on the spatial cognitive performance, productive performance and stress response in ewes, aiming to provide a theoretical basis for the application of music in welfare breeding to improve the cognitive function of ewes, thus improving the production performance and welfare of ewes.

## CONCLUSION

The music could affect the spatial reversal cognitive performance, growth performance and psychological response of ewes. The music of “Annie’s Wonderland” (55 dB) could improve the spatial cognitive performance, increase the ADG and lower the cortisol secretion of ewes, while the music of “Days of Youth Waltz” (55 dB) was not recommended because it might negatively impact the welfare of ewes.

## Figures and Tables

**Table 1:** Effects of music on the growth performance of ewes (mean ± S.E.)

Item	Control	“Annie’s Wonderland”	“Days of Youth Waltz”
ADFI (g/d)	1,028±59^a^	1,115±52^b^	943±65^c^
ADG (g/d)	289±17^a^	339±31^b^	241±18^c^
F/G	3.56±0.07^a^	3.29±0.06^b^	3.91±0.09^c^

a–c Different lowercase superscripts in the same line means significant difference (p<0.05).

SE, standard error; ADFI, average daily feed intake; ADG, average daily gain; F/G, feed/gain.

## SUPPLEMENTARY MATERIAL

**Supplement 1**. Effects of music types on the performance of the total time taken and the number of choice errors (mean ± S.E.; N=10) made in the Y-spatial test

**Supplement 2**. Effects of music types on the performance of the total time taken and the number of choice errors (mean ± S.E; N=8) made in the Y-spatial reversal test
