# Supplementary material for: Effects of music on the spatial cognitive performance, growth performance and stress response of sheep
Source: Anim Biosci. 2025 Mar 31;38(7):1543–56. doi: 10.5713/ab.24.0416 (PMC12229935; doi:10.5713/ab.24.0416)
Supplement: Supplementary file 1 [file ab-24-0416-Supplementary-1.pdf]

Supplement 1. Effects of music types on the performance of the total time taken and the number of choice errors (mean ±S.E.; N=10) made in the Y-spatial test.

Trail	Time (s)			Number of choice errors		
	Control	"Annie's Wonderland"	"Days of Youth Waltz"	Control	"Annie's Wonderland"	"Days of Youth Waltz"
1	46.95 ±2.55^{aA}	44.90 ±3.05^{aA}	60.98 ±3.23^{aB}	3.00 ±0.15^{a}	2.83 ±0.18^{a}	3.33 ±0.08^{a}
2	35.33 ±1.21^{bA}	32.94 ±2.25^{bA}	48.19 ±3.17^{bB}	2.33 ±0.20^{ab}	2.03 ±0.14^{a}	2.83 ±0.20^{ab}
3	40.84 ±3.20^{ab}	34.01 ±2.90^{b}	46.86 ±3.95^{b}	1.63 ±0.11^{bc}	1.30 ±0.10^{ab}	1.83 ±0.17^{bc}
4	36.16 ±2.50^{bA}	29.67 ±1.97^{bA}	48.97 ±3.85^{bB}	1.33 ±0.10^{bc}	1.00 ±0.20^{b}	1.67 ±0.13^{bc}
5	31.90 ±2.82^{bA}	30.41 ±2.83^{bA}	43.10 ±3.52^{bB}	1.00 ±0.20^{c}	0.80 ±0.08^{bc}	1.33 ±0.10^{c}
6	13.41 ±2.09^{cdA}	10.04 ±1.10^{cA}	24.98 ±1.95^{cB}	0.33 ±0.16^{d}	0.33 ±0.06^{cd}	0.33 ±0.04^{d}
7	16.07 ±1.40^{cA}	10.00 ±0.90^{cA}	26.05 ±2.10^{cB}	0.33 ±0.12^{d}	0.17 ±0.03^{d}	0.33 ±0.10^{d}
8	8.17 ±1.01^{cd}	8.63 ±0.78^{c}	11.88 ±1.05^{d}	0.00 ±0.00^{e}	0.00 ±0.00^{e}	0.17 ±0.05^{d}
9	11.35 ±1.13^{cd}	9.00 ±1.70^{c}	10.55 ±1.20^{d}	0.00 ±0.00^{e}	0.00 ±0.00^{e}	0.00 ±0.00^{e}
10	9.47 ±0.35^{d}	7.66 ±1.21^{cd}	6.42 ±0.73^{e}	0.00 ±0.00^{e}	0.00 ±0.00^{e}	0.00 ±0.00^{e}
11	7.29 ±1.21^{d}	6.47 ±0.30 ^{cd}	8.21 ±1.22^{de}	0.00 ±0.00^{e}	0.00 ±0.00^{e}	0.00 ±0.00^{e}
12	6.23 ±0.22^{de}	4.89 ±0.60^{d}	7.25 ±0.70^{de}	0.00 ±0.00^{e}	0.00 ±0.00^{e}	0.00 ±0.00^{e}
13	8.55 ±0.45^{de}	6.30 ±0.55^{cd}	9.23 ±0.79^{de}	0.00 ±0.00^{e}	0.00 ±0.00^{e}	0.00 ±0.00^{e}
14	6.13 ±0.55^{de}	5.30 ±0.57^{cd}	6.30 ±0.65^{e}	0.00 ±0.00^{e}	0.00 ±0.00^{e}	0.00 ±0.00^{e}
15	5.22 ±0.45^{e}	5.65 ±0.55^{cd}	6.59 ±0.75^{de}	0.00 ±0.00^{e}	0.00 ±0.00^{e}	0.00 ±0.00^{e}

Different capital letters in the same line means significant difference ($p<0.05$), different small letters in the same column means significant difference ($p<0.05$)
