# Supplementary material for: Effects of music on the spatial cognitive performance, growth performance and stress response of sheep
Source: Anim Biosci. 2025 Mar 31;38(7):1543–56. doi: 10.5713/ab.24.0416 (PMC12229935; doi:10.5713/ab.24.0416)
Supplement: Supplementary file 2 [file ab-24-0416-Supplementary-2.pdf]

Supplement 2. Effects of music types on the performance of the total time taken and the number of choice errors (mean ±S.E; N=8) made in the Y-spatial reversal test.

Trail	Time (s)			Number of choice errors		
	**Control**	**"Annie's Wonderland"**	**"Days of Youth Waltz"**	**Control**	**"Annie's Wonderland"**	**"Days of Youth Waltz"**
1	71.05±5.89^{aA}	65.98±6.21^{aA}	91.55±7.90^{aB}	5.33±0.43 ^{aA}	5.33±0.20^{aA}	6.67±0.23^{aB}
2	61.21±5.10^{abA}	55.60±4.91^{aA}	81.41±7.05^{abB}	5.33±0.43^{aA}	3.33±0.23^{bB}	5.67±0.30^{abA}
3	55.10±5.15^{bA}	47.15±4.00^{abA}	75.77±6.01^{bB}	3.33±0.21^{bA}	2.30±0.16^{bA}	5.33±0.43^{bB}
4	44.86±3.81^{cA}	36.20±3.03^{bcA}	65.70±4.75^{bcB}	1.53±0.12^{cdA}	0.83±0.11^{cA}	3.30±021^{cB}
5	32.22±2.03^{cdA}	23.35±1.70^{cdA}	50.70±4.00^{cB}	1.33±0.10^{cdA}	0.67±0.25^{cA}	3.33±0.23^{cB}
6	26.54±2.53^{de}	22.80±2.73^{cd}	30.89±3.01^{d}	0.83±0.11^{de}	0.67±0.25^{c}	1.17±0.12^{d}
7	27.15±2.01^{de}	22.50±1.95^{cd}	27.93±1.90^{de}	0.83±0.11^{de}	0.67±0.25^{c}	1.17±0.12^{d}
8	20.37±2.04^{de}	16.69±1.85^{d}	24.89±2.00^{de}	0.50±0.19^{e}	0.00±0.00^{d}	0.83±0.10^{d}
9	15.53±1.20^{e}	12.27±0.40^{e}	19.06±1.80^{de}	0.50±0.19^{e}	0.00±0.00^{d}	0.83±0.13^{d}
10	7.45±0.70^{efA}	6.85±0.66^{fA}	18.75±0.80^{deB}	0.00±0.00^{f}	0.00±0.00^{d}	1.33±0.21^{d}
11	9.33±0.21^{efA}	5.50±0.61^{fA}	20.00±1.65^{deB}	0.00±0.00^{f}	0.00±0.00^{d}	1.17±0.11^{d}
12	8.07±0.58^{efA}	5.60±0.45^{fA}	16.83±1.23^{efB}	0.00±0.00^{f}	0.00±0.00^{d}	1.17±0.21^{d}
13	8.30±0.79^{efA}	7.25±0.90^{efA}	17.25±1.10^{efB}	0.00±0.00^{f}	0.00±0.00^{d}	0.83±0.13^{d}
14	7.00±1.05^{fA}	5.85±1.34^{fA}	16.09±1.68^{efB}	0.00±0.00^{f}	0.00±0.00^{d}	0.83±0.10^{d}
15	6.43±0.73^{f}	5.00±0.15^{f}	9.00±1.24^{f}	0.00±0.00^{f}	0.00±0.00^{d}	0.00±0.00^{e}
16	6.37±0.30^{f}	5.37±0.49^{f}	8.32±2.01^{f}	0.00±0.00^{f}	0.00±0.00^{d}	0.00±0.00^{e}
17	6.69±0.50^{f}	5.77±0.58^{f}	7.95±0.75^{f}	0.00±0.00^{f}	0.00±0.00^{d}	0.00±0.00^{e}
18	7.91±1.90^{ef}	8.15±0.99^{ef}	8.08±0.80^{f}	0.00±0.00^{f}	0.00±0.00^{d}	0.00±0.00^{e}
19	5.61±1.01^{f}	7.60±0.59^{ef}	8.20±1.03^{f}	0.00±0.00^{f}	0.00±0.00^{d}	0.00±0.00^{e}
20	7.22±0.73^{f}	5.30±0.63^{f}	8.00±0.80^{f}	0.00±0.00^{f}	0.00±0.00^{d}	0.00±0.00^{e}

Different capital letters in the same line means significant difference (p<0.05), different small letters in the same column means significant difference (p<0.05).
